# Endometrial dysfunction in embryo implantation: from molecular mechanisms to clinical management

**DOI:** 10.3389/frph.2026.1865059

**Published:** 2026-06-18

**Authors:** Chang Shi, Ying Zhao, Wei Liu, Runqi Gong, Yinan Duan, Wenli Yu

**Affiliations:** 1 Department of Gynecology, Liaoning Maternal and Child Health Hospital, Shenyang, China; 2 Central Laboratory, Liaoning Maternal and Child Health Hospital, Shenyang, China

**Keywords:** embryo implantation, endometrial dysfunction, endometrial receptivity, recurrent implantation failure, thin endometrium

## Abstract

Endometrial dysfunction represents a central pathological factor underlying recurrent implantation failure and infertility. This review provides a comprehensive synthesis of the physiological basis of endometrial function, the pathological factors compromising its integrity, and the impact of these abnormalities on embryo implantation. Key pathogenic drivers, including cellular senescence, chronic inflammation, iatrogenic injury, endometriosis, and microbiome dysbiosis, converge on common mechanistic pathways such as decidualization impairment, immune dysregulation, epigenetic alterations, and mechanosensing defects. Building upon this mechanistic framework, we summarize current therapeutic interventions based on published literature. Available evidence suggests that intrauterine platelet-rich plasma (PRP) infusion appears promising among evaluated adjunctive interventions, but its definitive clinical superiority remains to be confirmed in large, standardized, head-to-head randomized trials. Granulocyte colony-stimulating factor (G-CSF), peripheral blood mononuclear cells (PBMCs), growth hormone, and stem cell-based therapies also show therapeutic potential but require further validation through well-designed studies. This review provides a theoretical foundation for understanding the association between endometrial dysfunction and embryo implantation failure and offers a practical, evidence-informed reference for personalized clinical treatment strategies.

## 1 Introduction

Globally, infertility affects a substantial proportion of reproductive-age couples. According to the most recent Global Burden of Disease study, the global age-standardized prevalence of female infertility was estimated at 3,713.2 per 100,000 population in 2021, corresponding to approximately 110 million affected women worldwide (1). Of these cases, 35% are attributable to female factors, 30% to male factors, 20% to combined factors, and 15% to unknown causes (2, 3). The decline in female fertility is closely associated with diminished ovarian function and endometrial dysfunction. Structural and functional abnormalities in the endometrium, including thin endometrium, endometrial senescence, and chronic endometritis, can impair endometrial receptivity through multiple mechanisms, such as altering glycosylation patterns, modulating the expression of receptivity-related genes, and enhancing local estrogen effects, thereby compromising embryo implantation and leading to infertility (4). Infertility caused by endometrial factors accounts for 10% of all infertility cases (5), posing a pervasive challenge for reproductive medicine specialists.

Despite significant advances in assisted reproductive technology (ART) over the past few decades and its success in treating many causes of infertility, embryo implantation after *in vitro* fertilization (IVF) remains challenging (6). Recurrent implantation failure resulting from inadequate endometrial development or dysfunction has emerged as a critical bottleneck limiting further improvements in ART outcomes.

Given this clinical context, this narrative review addresses two primary research questions: (i) What are the key molecular and cellular mechanisms by which endometrial dysfunction impairs embryo implantation? (ii) What is the current state of evidence for available therapeutic interventions aimed at improving endometrial function and pregnancy outcomes?

To address these questions, we offer three distinct contributions: (i) an integrated framework identifying shared pathogenic drivers (cellular senescence, chronic inflammation, iatrogenic injury, endometriosis, and microbiome dysbiosis) that converge on common mechanistic endpoints; (ii) integration of mechanistic insights with clinical translation, providing a practical reference for evidence-informed decision-making; and (iii) an overview of therapeutic strategies based on published network meta-analyses, with explicit discussion of evidence limitations.

## 2 Search strategy and evidence synthesis

To prepare this narrative review, we performed a targeted literature search using PubMed and Web of Science, focusing on publications from January 2000 to March 2026 to cover both foundational and recent advances. Search keywords included: endometrial dysfunction, endometrial receptivity, recurrent implantation failure, thin endometrium, embryo implantation, cellular senescence, chronic endometritis, microbiome, platelet-rich plasma, G-CSF, growth hormone, stem cell therapy, and their combinations. Two authors (C.S. and W.L.) independently screened titles and abstracts and reviewed reference lists of relevant articles to identify additional publications. We focused on full-text English articles from peer-reviewed journals, with emphasis on systematic reviews, meta-analyses, and randomized controlled trials where available. Observational studies and case series were included only for interventions with limited published data (e.g., stem cell therapy). When conflicting evidence was identified, we reported both findings and noted the discrepancy, giving greater consideration to more recent, larger, or methodologically robust studies.

## 3 Physiological function of the endometrium: from structural foundation to functional status

The endometrium is a dynamic tissue that undergoes continuous remodeling in response to ovarian steroid hormones. Throughout a woman's reproductive lifespan, the endometrium undergoes approximately 400 cycles of proliferation, differentiation, shedding, and regeneration (7). This complex, multicellular steroid-responsive tissue comprises stromal, epithelial, vascular, and immune cells (8). The endometrium exhibits significant morphological and physiological changes throughout the menstrual cycle, which are crucial for embryo implantation and successful pregnancy (9).

### 3.1 Tissue architecture and functional compartmentalization

The endometrium consists of a functional layer and a basal layer. The functional layer, which accounts for the upper two-thirds of the endometrium, comprises columnar epithelium lining the lumen and a multicellular stroma. This stroma includes fibroblastic stromal cells of connective tissue, spiral arteries, tissue-resident endometrial immune cells (including uNK cells, macrophages, and dendritic cells), and the periodic traffic of innate immune cells (10–12). The functional layer responds to ovarian hormones, undergoing dynamic changes in cell morphology and function. It sheds monthly in the absence of fertilization and serves as the site for embryo implantation. The basal layer, located below the functional layer, primarily regenerates the endometrium after menstruation (13). Recent single-cell sequencing studies have identified epithelial and mesenchymal subpopulations with stem/progenitor cell properties within the basal layer (14, 15).

### 3.2 Hormonal regulation of cyclic dynamics

Endometrial development and maturation are driven primarily by cyclic fluctuations in estrogen and progesterone. The synchronized regulation of the ovarian-endometrial axis involves the following sequence: ovarian follicles progress from primordial stages through to ovulation, accompanied by a peak in estrogen secretion; after ovulation, the corpus luteum forms and secretes both estrogen and progesterone; and in response to these hormonal signals, the endometrium undergoes cyclic proliferation, secretion, and shedding. Following menstruation, the proliferative phase is characterized by endometrial growth and re-epithelialization, driven mainly by rising estradiol levels from the dominant follicle. During this phase, extensive cellular proliferation occurs across all major endometrial cell types, including epithelial, stromal, and vascular cells. Peak proliferation, observed between cycle days 8 and 10, coincides with maximal estrogen receptor expression in the endometrium. After ovulation, the endometrium responds to estradiol and progesterone secreted by the corpus luteum. During the secretory phase, endometrial thickness remains relatively stable despite sustained elevated estradiol levels, an effect attributable in part to progesterone, which inhibits endometrial growth by reducing mitosis and DNA synthesis (16). In the absence of pregnancy, luteal regression triggers a sharp decline in progesterone and estradiol concentrations, initiating menstruation: spiral arteriole vasoconstriction induces distal ischemia, lysosomal enzyme release, and activation of matrix metalloproteinases, ultimately leading to apoptosis and disintegration of the functional layer, which is subsequently shed (17).

### 3.3 Regenerative capacity and functional reserve

The endometrium possesses one of the most robust regenerative capacities among human tissues, enabling rapid, scarless, and repetitive reconstruction. Endometrial epithelial progenitor cells and mesenchymal stem cells derived from the basal layer undergo hormone-directed migration and differentiation, achieving complete functional layer restoration within 4–5 days after menstruation (18). Recent studies have revealed that Wnt/β-catenin, Notch, and Hippo signaling pathways exhibit spatiotemporal regulation during endometrial regeneration and are tightly coupled with the decidualization process (19–21). Disruption of this regenerative program, whether due to mechanical injury (e.g., repeated intrauterine procedures), chronic inflammation, or microbial dysbiosis, can manifest as endometrial scarring (Asherman's syndrome), persistent thin endometrium, or defective functional layer differentiation, ultimately compromising embryo implantation (22–24).

### 3.4 The multidimensional shift in functional evaluation

Historically, assessment of endometrial function has relied heavily on morphological parameters—including thickness, echogenicity, and histological dating—as well as immunohistochemical detection of a limited set of molecular markers (25, 26). However, a growing consensus over the past five years has shifted the understanding that morphological integrity does not equate to functional competence. “Functional dimensions” such as decidualization potential (27), immune microenvironment homeostasis (28), cellular mechanosensing (29), and epigenetic temporal regulation (30) are progressively being integrated into frameworks for evaluating endometrial receptivity. This paradigm shift, moving from structural description toward functional state definition, provides a theoretical foundation for investigating the molecular mechanisms underlying endometrial dysfunction and for developing targeted interventions.

## 4 Factors affecting the physiological function of the endometrium

The maintenance of normal endometrial physiological function relies on the synergistic action of precise hormonal regulation, cellular homeostasis, and microenvironmental stability. When this balance is disrupted, multiple pathological factors can impair endometrial receptivity through shared molecular pathways, ultimately leading to recurrent implantation failure (RIF), recurrent pregnancy loss (RPL), and adverse pregnancy outcomes. To help readers understand the clinical relevance of the key mechanisms, we indicate the level of evidence according to the Oxford Centre for Evidence-Based Medicine (OCEBM) (31) in Table 1.

**Table 1:** Evidence levels of key mechanisms underlying endometrial dysfunction based on current literature using the 2011 OCEBM guidelines.

Mechanism	Key evidence type	Oxford level
SASP (senescence-associated secretory phenotype)	Human tissue (case-control), *in vitro*	Level 3
TGF-β/Smad	Human tissue (case-control), *in vitro*, animal	Level 3
YAP/TAZ	*In vitro* (human primary cells), animal	Level 4
Progesterone resistance	Human cohort, case-control, *in vitro*	Level 2
HOXA10 methylation	Human tissue (case-control), *in vitro*	Level 3
NF-*κ*B activation	Human tissue (case-control), *in vitro*	Level 3
Microbiome-derived metabolites	Human association (case-control), *in vitro*	Level 3
Epigenetic regulation	Human tissue (case-control), *in vitro*, animal	Level 3

### 4.1 Cellular senescence and inflammatory microenvironment remodeling

Cellular senescence plays a dual role in both physiological renewal and pathological disruption of the endometrium. Under physiological conditions, senescent cells contribute to menstrual tissue shedding and subsequent regeneration. In contrast, pathological accumulation of senescent cells drives chronic inflammation and promotes tissue fibrosis (32, 33). Yan et al. systematically characterized the dual functions of endometrial senescence: under physiological conditions, senescent cells support tissue repair through the secretion of specific factors; however, under pathological conditions, excessive activation of the senescence-associated secretory phenotype (SASP) leads to dysregulated decidualization and aberrant expression of vascular endothelial growth factor receptors, ultimately compromising endometrial receptivity (32). Tamura et al. further demonstrated in the context of endometriosis that SASP establishes a positive feedback loop with prostaglandin metabolism disorders. Specifically, senescent cells accumulated in ectopic lesions release pro-inflammatory factors via SASP, which on one hand exacerbates local inflammation and on the other hand interferes with progesterone signal transduction in eutopic endometrial stromal cells (34). Impaired immune clearance of senescent cells has recently emerged as a novel therapeutic target for endometrial disorders.

### 4.2 Iatrogenic injury and fibrosis

Repeated intrauterine procedures (including induced abortion, hysteroscopic surgery, and diagnostic curettage) constitute the most common iatrogenic causes of endometrial damage (35). Mechanical injury triggers a local inflammatory response, activating the TGF-β/Smad signaling pathway, which in turn promotes fibroblast activation and excessive extracellular matrix deposition. This cascade ultimately leads to endometrial fibrosis and scar formation, the fundamental pathological basis of Asherman's syndrome (36). Recent studies have demonstrated that mechanical stress itself can directly regulate the differentiation of fibroblasts into myofibroblasts through the YAP/TAZ signaling pathway, thereby driving the fibrotic process independently of inflammatory mediators (37). This finding provides insight into why some patients may develop progressive endometrial fibrosis following repeated intrauterine procedures, even in the absence of overt evidence of infection.

### 4.3 Endometriosis: progesterone resistance and epigenetic abnormalities

The eutopic endometrium of patients with endometriosis exhibits intrinsic functional defects. Tamura et al. demonstrated that endometrial stromal cells from patients with endometriosis display progesterone resistance, characterized by an imbalance in progesterone receptor isoforms, abnormal expression of co-regulators, and downregulation of downstream target genes including HOXA10 and IGFBP-1 (34). Pîrlog et al. systematically reviewed the roles of HOXA10 and HOXA11, noting that hypermethylation of the HOXA10 promoter region in the endometrium of patients with endometriosis leads to gene silencing, thereby affecting the expression of key receptivity molecules such as integrin β3 and LIF (38). Furthermore, endometriosis-associated endometrial dysfunction exhibits molecular subtype heterogeneity: some cases are predominantly characterized by impaired decidualization (CMA type), whereas others are marked by upregulation of immune-related genes (BMA type), which has guiding significance for treatment strategy selection (39).

### 4.4 Chronic endometritis and microbiome dysbiosis

Chronic endometritis (CE) is a persistent, low-grade inflammatory condition marked by the infiltration of plasma cells (CD138⁺) in the endometrial stroma (40). Traditionally, CE was viewed as a consequence of persistent pathogen infection; however, recent studies have revealed greater pathological complexity. Yan et al. pointed out that even after successful pathogen clearance with antibiotics, some patients exhibit persistent infiltration of CD138⁺ cells, which have transitioned to a pro-fibrotic, pro-senescent secretory phenotype, suggesting that CE is essentially a disorder of immune microenvironmental memory (41).

Advances in endometrial microbiome research have reshaped our understanding of CE pathogenesis. Stoyancheva et al. emphasized that the endometrium is not a sterile environment but instead harbors functionally active, low-biomass microbial communities (42). A Lactobacillus-dominant microbial state is associated with endometrial homeostasis and favorable reproductive outcomes, whereas dysbiosis, characterized by increased microbial diversity and enrichment of anaerobic bacteria such as Gardnerella, Atopobium, and Streptococcus, is closely linked to CE, implantation failure, and adverse IVF outcomes. Host-microbiome interactions encompass multiple mechanistic levels: i) Immune modulation: pathogen-associated molecular patterns continuously activate NF-*κ*B via TLR4, thereby interfering with progesterone signal transduction; ii) Metabolic regulation: short-chain fatty acids produced by lactobacilli help maintain a chromatin state permissive for receptivity-associated gene expression through HDAC inhibition; and iii) Epithelial barrier function: tryptophan metabolites (e.g., indole-3-propionic acid) upregulate IL-22 expression and tight junction proteins via activation of the aryl hydrocarbon receptor (42, 43).

## 5 Effects of endometrial receptivity on embryo implantation

Embryo implantation is the process by which a blastocyst adheres to and invades the endometrium, initiating the formation of the maternal-fetal interface. This process requires coordinated communication between a viable embryo and a receptive endometrium. The term “implantation window” refers to the limited period during the secretory phase of the menstrual cycle when the endometrium can accept blastocysts, typically between Days 20–24 of a standard ovulatory menstrual cycle in humans (44). Implantation involves complex biological mechanisms regulated by hormones such as estrogen and progesterone, as well as cytokines, growth factors, and immune regulators (45–47). Together, these elements drive the morphological and molecular changes that establish optimal conditions for successful blastocyst–endometrial interaction.

### 5.1 The concept and assessment of endometrial receptivity

Endometrial receptivity refers to the capacity of the endometrium to “accept” embryo implantation within a specific temporal window, following morphological and functional changes induced by ovarian hormones, thereby creating an optimal environment for the establishment and progression of pregnancy (48). This receptive state is typically most favorable during the mid-secretory phase, coinciding with oocyte maturation, fertilization, and embryo implantation. Outside this window, the endometrium becomes progressively less receptive, making successful implantation increasingly difficult to achieve (8). Approximately two-thirds of embryo implantation failures are attributable to impaired endometrial receptivity and inadequate embryo-endometrial dialogue, whereas embryo quality accounts for only one-third of such failures (26).

Over the past decade, assessment of endometrial receptivity has gained increasing prominence in evaluating the endometrial contribution to pregnancy success. Although various methods and techniques for evaluating receptivity have become increasingly available, noninvasive ultrasound examination remains the most widely used approach in clinical practice (49). This evaluation typically encompasses endometrial pattern classification, assessment of uterine artery and subendometrial blood flow, and measurement of uterine cavity volume (50). Among these parameters, endometrial thickness has emerged as the most commonly employed clinical indicator of receptivity, owing to its ease of measurement and high reproducibility (51). Endometrial thickness is primarily regulated by estrogen and its receptor expression: during the proliferative phase, estrogen stimulation drives endometrial cell proliferation and rapid tissue expansion, representing the critical period for determining endometrial thickness (52). Numerous studies have demonstrated that endometrial thickness is closely associated with pregnancy outcomes in both natural conception cycles and IVF treatment cycles (53, 54). However, endometrial thickness is a surrogate marker; improvements in thickness do not necessarily translate into improved live birth rates.

### 5.2 Thin endometrium and implantation failure

In clinical practice, thin endometrium (TE) is widely recognized as a frequent contributor to impaired endometrial receptivity (55). Successful embryonic attachment and invasion during the mid-secretory phase depend on effective communication among endometrial cells and between the endometrium and the embryo. However, these signaling networks are often disrupted in patients with TE (56). TE may result from multiple factors, including mechanical damage to the endometrium, slow glandular epithelial growth, elevated uterine artery blood flow resistance, reduced levels of vascular endothelial growth factor (VEGF), and diminished estrogen receptor expression (57). These causes most commonly arise from inadequate endometrial repair following intrauterine adhesion surgery, which can lead to vascular disruption and glandular sparsity (58). Several studies have demonstrated that clinical pregnancy, implantation, and live birth rates are significantly reduced in patients with TE (59), and TE has also been associated with adverse perinatal outcomes (60).

The endometrium of patients with TE often exhibits pathological features such as decreased estrogen receptor expression (61), impaired vascular development (62), increased blood flow resistance (63), and elevated oxygen partial pressure in the functional layer (23, 63). Endometrial and ovarian blood perfusion in TE patients is lower than in those with normal endometrium (64). This hypoperfusion results in reduced endometrial VEGF expression and restricted endometrial growth (65). Studies have shown that low oxygen concentrations can promote embryo implantation (66). In patients with TE, implantation is challenging because the embryo lies in closer proximity to areas with high oxygen tension. Research has also indicated that TE is a risk factor for ectopic pregnancy following IVF and embryo transfer (67). This may be related to the embryo's tendency to migrate to extrauterine sites with lower oxygen tension within a narrowed uterine cavity (68).

### 5.3 The threshold controversy of endometrial thickness

The optimal endometrial thickness for achieving receptivity remains a subject of debate. Generally, an endometrial thickness of 7 mm during the mid-secretory phase is considered suggestive of adequate receptivity (54, 69–72), although some studies propose cutoff values of 8 mm or even 6 mm (60, 73, 74). A large Canadian study encompassing 43,383 fresh and 53,377 frozen embryo transfer cycles reported that live birth rates (LBR) in fresh cycles were optimized when endometrial thickness measured 10–12 mm. In frozen-thawed cycles, LBR plateaued at thicknesses between 7 and 10 mm, whereas thicknesses below 6 mm were significantly associated with reduced LBR (74). A study by Ata et al. involving 959 single euploid blastocyst transfers similarly found that the group with endometrial thickness of 10–12 mm achieved higher LBR, although the difference did not reach statistical significance when compared to the thinner endometrium group (75).

The etiology of extremely TE is often unclear and may not be readily correctable. Numerous retrospective studies have evaluated the feasibility of successful embryo transfer in the setting of minimal endometrial thickness, yielding inconsistent results. Successful pregnancies with endometrial thickness below 4 mm have been rarely documented (76), and the thinnest endometrial thickness associated with a healthy term live birth following assisted reproduction was reported to be 3.5 mm (77). Collectively, these uncertainties highlight the inherent limitations of relying solely on endometrial thickness as a marker of receptivity. A more precise evaluation requires integrating parameters such as blood flow characteristics, molecular markers, and functional dimensions of endometrial competence.

## 6 Methods for improving endometrial function and pregnancy rate

Thin endometrium and diminished endometrial receptivity represent long-standing clinical challenges in assisted reproduction. Multiple therapeutic strategies have been applied, including hormonal regulation, vasoactive drugs, intrauterine infusions, physical therapies, and regenerative medicine approaches. However, the strength of evidence supporting different interventions varies considerably, and clinical decision-making often lacks a transparent, evidence-informed foundation.

As detailed in Section 2, this narrative review summarizes evidence from published systematic reviews, meta-analyses, and randomized controlled trials (RCTs), supplemented by observational studies where published data are limited. The evidence for each intervention is discussed with attention to the methodological limitations described throughout this section.

The studies reviewed here encompass several distinct patient groups, including women with thin endometrium, RIF, unexplained infertility, endometriosis-associated infertility, and donor oocyte recipient cycles. These populations differ substantially in underlying pathophysiology, baseline endometrial function, and prognosis. Most therapeutic studies have focused on thin endometrium or RIF, whereas evidence for other groups (e.g., endometriosis without documented thin endometrium) is more limited. Thus, extrapolation of findings from one population to another requires caution. Where possible, the population studied is specified for each intervention; when evidence is lacking for specific subgroups, this is noted as a limitation.

Before evaluating individual interventions, a conceptual note is also warranted: endometrial thickness is a surrogate endpoint commonly reported in therapeutic studies. While increased thickness may reflect improved endometrial proliferation, it does not guarantee enhanced receptivity or live birth.

### 6.1 Hormonal regulation and endometrial proliferation strategies

#### 6.1.1 Estrogen

Estrogen supplementation is a foundational approach for improving endometrial thickness. Prolonging the duration of estrogen exposure or optimizing the route of administration has been explored in patients with poor endometrial response in hormone replacement therapy (HRT) cycles. A systematic review and meta-analysis including 7 RCTs with 898 patients undergoing frozen-thawed embryo transfer (FET) demonstrated that the choice of estrogen formulation may impact endometrial thickness: the transdermal route was associated with a slight but significant increase in endometrial thickness compared to oral estrogen (SMD = 0.16; 95% CI 0.02–0.31) (78). However, no significant differences were observed in clinical pregnancy rates (OR = 0.98; 95% CI 0.74–1.31), implantation rates, or live birth rates between different estrogen administration routes (78). A separate prospective RCT (*n* = 82) comparing transdermal gel versus oral estrogen confirmed no significant differences in endometrial thickness, biochemical pregnancy rates, or clinical pregnancy rates (79).

The evidence consistently shows that while the transdermal route may offer a marginal advantage in endometrial thickness, this does not translate into improved pregnancy outcomes. Key limitations include heterogeneity in estrogen formulations, treatment duration, and patient populations across the included RCTs. Most studies were not powered to detect differences in live birth rates. For the general IVF population, the specific route of estrogen administration appears to have limited impact on ultimate pregnancy success. Extended estrogen exposure has been used empirically for patients with thin endometrium, but robust evidence for this strategy is lacking (80).

#### 6.1.2 Growth hormone (GH)

GH promotes endometrial proliferation and angiogenesis by upregulating insulin-like growth factor-1 (IGF-1) and vascular endothelial growth factor (VEGF) expression (81). Network meta-analyses have demonstrated that intramuscular injection of recombinant human GH is associated with increased endometrial thickness, ranking favorably among six commonly used interventions for thin endometrium (82); it has also been associated with improved clinical pregnancy rates compared to controls (OR = 1.73, 95% CI 1.02–2.94) (83). However, a recent RCT in donor oocyte recipient cycles found that GH administration from day 2–12 of the cycle resulted in comparable endometrial thickness (9.30 mm vs. 9.18 mm, *P* > 0.05) and clinical pregnancy rates (78% vs. 76%, *P* > 0.05) compared to controls, suggesting that the benefit of GH may be limited to specific populations, such as those with refractory thin endometrium, rather than unselected patients (84).

The evidence for GH is characterized by marked population-dependent heterogeneity. Positive findings come primarily from studies enrolling patients with thin endometrium or prior implantation failure, whereas a recent well-controlled trial in healthy donor oocyte recipients found no benefit. This discrepancy suggests that GH has been studied primarily as a rescue intervention in selected patients with endometrial dysfunction, whereas routine use in all ART cycles is not supported by current evidence. Limitations include small sample sizes in most positive studies, variability in dosing regimens (daily vs. every-other-day, starting day of cycle), and lack of long-term safety data. Studies reporting positive outcomes have focused on patients with refractory thin endometrium or prior implantation failure; a negative trial in healthy donor oocyte recipients suggests that the benefit may not extend to unselected populations.

### 6.2 Pharmacological interventions to improve endometrial blood flow

#### 6.2.1 Aspirin

Aspirin exerts its therapeutic effects through multiple mechanisms, including inhibition of platelet aggregation, reduction of vasoconstriction via cyclooxygenase suppression and decreased thromboxane A₂ production, thereby improving uterine artery blood flow (85). According to a network meta-analysis of six interventions for thin endometrium, aspirin ranked second for improving endometrial thickness (SUCRA = 70.89%) and second for clinical pregnancy rates (SUCRA = 70.29%) among the evaluated interventions (82). However, these rankings are derived from indirect evidence, and direct comparisons with other active interventions are lacking. Furthermore, another network meta-analysis also reported a significant improvement in clinical pregnancy rates with aspirin compared to controls (OR = 1.87, 95% CI 1.06–3.29) (83).

Aspirin has consistently ranked among the top interventions for improving both endometrial thickness and clinical pregnancy rates in network meta-analyses. Nevertheless, the effect size is modest, and the confidence interval indicates some imprecision. Most studies focused on surrogate outcomes (endometrial thickness) rather than live birth, and the optimal dosing regimen (typically 50–100 mg daily) and treatment duration remain unspecified. Aspirin has been evaluated as an adjunctive treatment in patients with thin endometrium or vascular insufficiency. Its low cost, wide availability, and favorable safety profile at low doses have made it a commonly studied intervention, particularly in settings where more targeted therapies are not accessible.

#### 6.2.2 Sildenafil

Sildenafil, a phosphodiesterase-5 (PDE5) inhibitor, selectively dilates the uterine arteries, increasing blood flow to the uterus and improving endometrial development (86). Several randomized controlled trials have evaluated its efficacy in improving endometrial thickness. Sarhan et al. demonstrated that oral sildenafil 20 mg twice daily significantly increased endometrial thickness in women with unexplained infertility undergoing clomiphene citrate stimulation (median ET: 8 mm vs. 7 mm in controls, *P* < 0.01) (87). Belapurkar et al. reported a mean increase in endometrial thickness of 3.87 mm with vaginal sildenafil 25 mg every six hours from cycle day 6–12 (88). A meta-analysis confirmed that sildenafil significantly improves endometrial thickness compared to controls (WMD = 1.66 mm, 95% CI 0.59–2.74) in women undergoing assisted reproductive technology (89). More recently, Bosenge-Nguma et al. conducted a double-blind RCT (*n* = 74 per group) comparing oral sildenafil plus clomiphene citrate versus estradiol valerate plus clomiphene citrate in women with unexplained infertility, reporting significantly higher Applebaum scores (17.05 vs. 15.14, *P* = 0.000) and clinical pregnancy rates (28.92% vs. 20.83%, *P* = 0.04) in the sildenafil group (90).

Sildenafil consistently improves endometrial thickness across multiple RCTs and meta-analyses. However, evidence supporting its impact on clinical pregnancy rates is less robust, as most studies were not powered for pregnancy outcomes. Substantial heterogeneity exists in dosing (oral vs. vaginal, 20–100 mg daily) and treatment duration, with no established optimal regimen. Sildenafil has been reported to improve endometrial thickness with an acceptable safety profile, but evidence supporting its effect on live birth remains limited. The available studies have primarily enrolled women with unexplained infertility or thin endometrium; evidence in RIF or endometriosis-associated dysfunction is lacking.

### 6.3 Intrauterine infusion therapies

#### 6.3.1 Platelet-rich plasma (PRP)

PRP is rich in multiple growth factors (PDGF, TGF-β, VEGF, IGF-1, EGF) and promotes endometrial proliferation, angiogenesis, and immunomodulation through autocrine and paracrine mechanisms. Since Chang et al. first reported the successful treatment of refractory thin endometrium with PRP intrauterine infusion in 2015 (91), multiple RCTs and systematic reviews have been published.

Several network meta-analyses have reported favorable outcomes associated with PRP. A network meta-analysis of 18 RCTs for thin endometrium reported that PRP had the highest ranking for clinical pregnancy rates (SUCRA = 80.12%) and the third highest for endometrial thickness (SUCRA = 68.14%) (82). Another network meta-analysis focusing on 3,035 patients with RIF reported that PRP had the highest rankings for both clinical pregnancy (SUCRA = 84.5%) and live birth rates (SUCRA = 81.4%) (92). A third network meta-analysis similarly reported clinical benefits of PRP (83). However, these rankings derive from indirect comparisons, and direct head-to-head trials between PRP and other active interventions are scarce.

Optimization strategies have been explored in recent RCTs. A trial by Feng et al. (*n* = 100) compared single versus double PRP infusion, finding that double infusion significantly improved endometrial thickness (8.42 vs. 7.96 mm, *P* < 0.01) and clinical pregnancy rates (48.9% vs. 27.0%, *P* = 0.043) (93). Another RCT by Zhang et al. reported that PRP combined with endometrial microstimulation further improved endometrial thickness compared to PRP alone (94).

The evidence for PRP is derived from multiple RCTs, meta-analyses, and network meta-analyses, with generally consistent findings across most systematic reviews. However, several limitations warrant caution. An umbrella review noted that the level of evidence varied from very low to high (more commonly low or moderate), primarily due to methodological flaws and clinical heterogeneity (95). Substantial variation exists in PRP preparation methods, infusion protocols, and patient selection criteria, limiting generalizability. Network meta-analyses rely heavily on indirect comparisons, and direct head-to-head trials are scarce. Therefore, while PRP appears promising among evaluated adjunctive interventions, its clinical superiority, particularly for live birth, has not been confirmed and awaits evaluation through large, standardized, head-to-head randomized trials.

#### 6.3.2 Granulocyte colony-stimulating factor (G-CSF)

G-CSF exerts its effects by recruiting endometrial stem cells, promoting angiogenesis, and modulating immune responses (96). According to a network meta-analysis of thin endometrium interventions, G-CSF had the highest ranking for increasing endometrial thickness (SUCRA = 78.48%) but a lower ranking for clinical pregnancy rates (SUCRA = 65.7%) compared to PRP (82). A network meta-analysis of RIF similarly reported that G-CSF improved pregnancy outcomes, with lower rankings than PRP and PBMCs (92). These rankings are based on indirect evidence; direct comparisons between G-CSF and other active interventions are limited.

The available evidence consistently supports the ability of G-CSF to increase endometrial thickness, but its impact on live birth remains unproven. Although statistically significant, the effect size for clinical pregnancy is modest, and G-CSF consistently ranks below PRP and PBMCs for pregnancy outcomes across network meta-analyses. Optimal dosing (intrauterine vs. subcutaneous, single vs. repeated) and patient selection criteria are not yet established. Thus, G-CSF has been evaluated as an alternative in clinical studies, particularly in settings where PRP is not available. The evidence for G-CSF comes from two distinct populations: thin endometrium (where it ranks highly for thickness) and RIF (where it ranks lower than PRP and PBMCs for pregnancy outcomes).

#### 6.3.3 Human chorionic gonadotropin (hCG)

Intrauterine infusion of hCG mimics early embryonic signals and modulates the local immune microenvironment (97). A network meta-analysis of RIF reported that hCG was associated with improvements in pregnancy outcomes, with SUCRA values of 52.5% for clinical pregnancy and 48.7% for live birth, which were the lowest among the evaluated intrauterine infusion therapies (92). As with other network meta-analysis findings, these rankings are based on indirect comparisons and should be interpreted cautiously.

hCG has been evaluated in multiple RCTs and meta-analyses, with evidence generally supporting its efficacy in RIF patients. However, among commonly used intrauterine infusion therapies, hCG consistently ranks lowest for both clinical pregnancy and live birth outcomes. This inferior ranking, together with the availability of more effective alternatives such as PRP and PBMCs, suggests that hCG has been studied less extensively in this context. Nevertheless, hCG is widely available, has a favorable safety profile, and is less costly compared to PRP or cell-based therapies. Most studies of intrauterine hCG have been conducted in RIF patients; evidence in thin endometrium is limited.

#### 6.3.4 Peripheral blood mononuclear cells (PBMCs)

Intrauterine infusion of PBMCs may improve endometrial receptivity by modulating the local immune microenvironment through cytokine and growth factor cascades (98). A network meta-analysis of RIF reported that PBMCs had the second highest rankings for clinical pregnancy (SUCRA = 76.5%) and live birth (SUCRA = 64.6%) among evaluated intrauterine infusion therapies, after PRP (92). However, these rankings are derived from a limited number of studies and rely on indirect evidence; direct comparisons with other interventions are lacking.

The evidence base for PBMCs is more limited than that for PRP, with relatively few RCTs and most studies originating from a limited number of research groups, raising concerns about generalizability. Nonetheless, PBMCs have been reported as an alternative in studies where PRP was not used. The available evidence is restricted almost exclusively to RIF populations; no studies have evaluated PBMCs specifically for thin endometrium.

### 6.4 Physical therapies

Pelvic floor neuromuscular electrical stimulation (NMES) improves pelvic blood flow and local microcirculation. A network meta-analysis of thin endometrium interventions reported that NMES ranked third for increasing endometrial thickness (SUCRA = 68.14%), following G-CSF and aspirin, although its effect on clinical pregnancy rates was limited (82). As with other findings from this network meta-analysis, the ranking is based on indirect evidence and should be interpreted cautiously. Other physical therapy modalities, such as transcutaneous electrical acupoint stimulation (TEAS) and low-intensity pulsed ultrasound (LIPUS), have also shown preliminary benefit in improving endometrial thickness and pregnancy rates, but evidence remains limited (99, 100).

The available evidence suggests that NMES may have a beneficial effect on endometrial thickness, with a favorable safety profile and low cost. However, data on pregnancy outcomes are lacking. NMES has been studied primarily in thin endometrium; evidence for its use in RIF is not available.

### 6.5 Stem cell therapy

Stem cell therapy has been investigated as a potential approach for regenerative repair in refractory thin endometrium and Asherman's syndrome. Mesenchymal stem cells (MSCs) derived from sources such as bone marrow, umbilical cord, adipose tissue, endometrium, and menstrual blood have been reported to promote tissue repair and angiogenesis primarily through paracrine mechanisms involving exosomes and growth factors (101, 102).

A systematic review summarized emerging biotechnologies for endometrial pathologies and noted that most clinical studies of stem cells remain at early stages, although the evidence to date supports the safety and feasibility of these approaches (103). A meta-analysis by Adamyan et al. including 18 clinical studies (323 patients) reported a mean increase in endometrial thickness of 2.35 mm after MSC treatment compared to baseline (95% CI 1.97–2.74, *P* < 0.00001) (104). Subgroup analysis suggested that endometrial-derived and adipose-derived MSCs were associated with the largest increases. An RCT subgroup analysis indicated significant improvements in clinical pregnancy rates (OR = 2.72, *P* = 0.002) and live birth rates (OR = 2.27, *P* = 0.01), as well as a reduction in miscarriage rates (OR = 0.24, *P* = 0.004) (103). Of note, the evidence for these estimates came predominantly from non-randomized or single-arm studies.

Stem cell therapy for endometrial dysfunction remains experimental. Prior to broader clinical application, several unresolved issues require attention: (i) absence of standardized cell sourcing, characterization, and manufacturing protocols; (ii) unknown long-term tumorigenic risk; (iii) lack of follow-up data on maternal and neonatal outcomes; (iv) high heterogeneity in cell sources, delivery routes (intrauterine infusion, transmyometrial injection, scaffold-assisted), and dosing regimens; and (v) high cost limiting accessibility. Given these limitations, stem cell therapy has been used only in clinical research settings with rigorous ethical and regulatory oversight, and it is not established for routine clinical application.

### 6.6 Emerging directions and future perspectives

#### 6.6.1 Drug repurposing strategies

Based on transcriptome-drug feature matching, already-approved drugs such as genistein, pioglitazone, and alprostadil are predicted to improve endometrial function, although clinical validation is pending (39).

#### 6.6.2 Induced pluripotent stem cells (iPSCs)

Differentiation into oocyte-like cells has been achieved in animal models, but remains at the preclinical stage (105, 106).

#### 6.6.3 Personalized treatment selection

Individualization of endometrial preparation based on patient characteristics has been proposed as a strategy to optimize reproductive outcomes (107). Precision therapy based on endometrial molecular subtyping (e.g., dysbiotic, inflammatory, or mixed endometrial dysfunction) represents a future direction, although rapid point-of-care testing tools are currently lacking (42).

### 6.7 Overall evidence limitations and summary

Several methodological limitations apply broadly across the literature on endometrial dysfunction interventions. Most RCTs are single-center with small sample sizes (typically 30–100 participants per arm), limiting statistical power for live birth outcomes. Substantial heterogeneity exists in treatment protocols, including dosing, timing, frequency, and patient selection, which complicates cross-study comparisons. Publication bias is a concern, as positive results are more likely to be published, particularly for novel interventions such as PRP and stem cell therapy. Most studies focus on surrogate endpoints (endometrial thickness, clinical pregnancy) rather than live birth, and long-term maternal or neonatal safety data are lacking. Direct head-to-head comparisons between active interventions are scarce; rankings from network meta-analyses rely heavily on indirect evidence and should be interpreted cautiously.

Among the interventions evaluated, intrauterine PRP infusion appears promising based on current evidence and may be considered for patients with thin endometrium or RIF. However, its evidence base remains constrained by protocol heterogeneity, indirect comparisons, and lack of long-term safety data. Definitive conclusions regarding its clinical superiority require confirmation through large, standardized, head-to-head randomized trials. G-CSF, PBMCs, and growth hormone have also been studied, although the supporting evidence is less extensive and more heterogeneous across studies. Stem cell therapy remains experimental and has been limited to clinical research settings; it has not been established for routine clinical application.

## 7 Conclusions and future perspectives

The physiological integrity of endometrial function constitutes a fundamental cornerstone of female reproductive health. Multiple pathological factors, including cellular senescence, chronic inflammation, iatrogenic injury, endometriosis, and microbiome dysbiosis, can impair endometrial receptivity through shared molecular pathways, ultimately leading to recurrent implantation failure and adverse pregnancy outcomes. This review has summarized these molecular mechanisms and provided an evidence-informed overview of current therapeutic strategies based on published literature.

Among available interventions, intrauterine PRP infusion appears promising based on published network meta-analyses, suggesting potential efficacy in thin endometrium and RIF. However, the overall evidence remains limited by protocol heterogeneity, indirect comparisons, and lack of long-term safety data. G-CSF, PBMCs, and growth hormone show therapeutic potential but require further validation. Stem cell-based therapies remain experimental and have been used only in research settings.

Future research may focus on several areas: (i) personalized strategies based on molecular subtyping; (ii) clinical translation and safety evaluation of stem cell and exosome-based therapies; and (iii) deeper understanding of endometrial regeneration mechanisms. Such efforts could help establish a foundation for precision management of endometrial dysfunction and ultimately improve female reproductive health and pregnancy outcomes.
